# Study protocol of an investigation of attention and prediction error as mechanisms of action for latent inhibition of dental fear in humans

**DOI:** 10.1186/s40359-023-01054-0

**Published:** 2023-01-25

**Authors:** Laura D. Seligman, Andrew L. Geers, Lauren Kramer, Kelly S. Clemens, Keenan A. Pituch, Ben Colagiuri, Hilary A. Marusak, Christine A. Rabinak, Natalie Turner, Michael Nedley

**Affiliations:** 1 Department of Psychological Science, University of Texas Rio Grande Valley, Edinburg, TX 78539 USA; 2 Department of Psychology, University of Toledo, Toledo, OH USA; 3 Edson College of Nursing and Health Innovation, Arizona State University, Phoenix, AZ USA; 4 Department of Psychology, University of Sydney, Sydney, Australia; 5 Department of Psychiatry and Behavioral Neuroscience, School of Medicine, Wayne State University, Detroit, MI USA; 6 Department of Pharmacy Practice, Wayne State University, Detroit, MI USA; 7 Department of Pediatric Dentistry, University of Toledo College of Medicine and Life Sciences, Toledo, OH USA

**Keywords:** Dental phobia, Latent inhibition, Fear learning, Pre-exposure, Pain sensitivity, Virtual reality, Eye tracking

## Abstract

**Background:**

Evidence suggests that dental anxiety and phobia are frequently the result of direct associative fear conditioning but that pre-exposure to dental stimuli prior to conditioning results in latent inhibition of fear learning. The mechanisms underlying the pre-exposure effect in humans, however, are poorly understood. Moreover, pain sensitivity has been linked to dental fear conditioning in correlational investigations and theory suggests it may moderate the latent inhibition effect, but this hypothesis has not been directly tested. These gaps in our understanding are a barrier to the development of evidence-based dental phobia prevention efforts.

**Methods:**

Healthy volunteers between the ages of 6 and 35 years will be enrolled across two sites. Participants will complete a conditioning task in a novel virtual reality environment, allowing for control over pre-exposure and the examination of behaviour. A dental startle (a brief, pressurized puff of air to a tooth) will serve as the unconditioned stimulus. Using a within-subjects experimental design, participants will experience a pre-exposed to-be conditioned stimulus, a non-pre-exposed to-be conditioned stimulus, and a neutral control stimulus. Two hypothesized mechanisms, changes in prediction errors and attention, are expected to mediate the association between stimulus condition and fear acquisition, recall, and retention. To ascertain the involvement of pain sensitivity, this construct will be measured through self-report and the cold pressor task.

**Discussion:**

Dental phobia negatively affects the dental health and overall health of individuals. This study aims to determine the mechanisms through which pre-exposure retards conditioned dental fear acquisition, recall, and retention. A randomized control trial will be used to identify these mechanisms so that they can be precisely targeted and maximally engaged in preventative efforts.

## Introduction

In the United States, dental fear and anxiety are prevalent issues that impact the oral health of 40–50 million Americans [1]. Moreover, Hispanic and African American populations are disproportionally impacted by poor oral health [2, 3], with dental fear as a common reason for avoiding dental visits in these populations [4]. A major etiological factor for dental fear is direct associative conditioning [5] but natural history studies suggest that pre-exposure to dental stimuli prior to a fear conditioning event can result in latent inhibition (LI), the retardation of associative conditioning as the result of prior learning [6, 7]. These findings suggest pre-exposure to dental stimuli as a method by which the development of dental fear and phobia could be prevented. However, the causal effect of LI on dental fear has not been established using experimental methods.

**Fig. 1:**
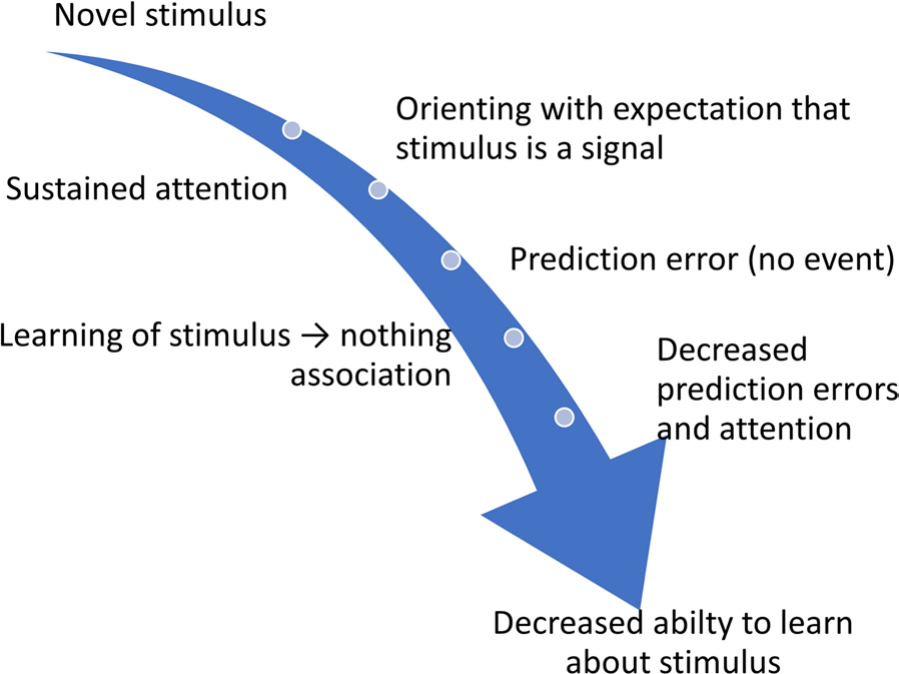
Hall and Rodriguez model

Moreover, to effectively harness the potential of LI to prevent dental fear, the underlying mechanism(s) must be better understood. Hall and Rodriguez ([8], see Fig. 1) have proposed a model of LI which starts with the well-established finding that a novel stimulus garners significant attention because of its potential to serve as a signal of a relevant event [9]. The model suggests that if the stimulus is not followed by a relevant event upon early presentations, a stimulus → no event association is learned. Over time, prediction errors (i.e., stimulus → event) are expected to decrease because of this learning, in turn, resulting in a decrease in attention as the lack of relevance of the stimulus is learned. If a pre-exposed stimulus is later paired with a relevant outcome (i.e., an unconditioned stimulus, UCS), this lack of attention renders the stimulus less available to enter an association with the UCS, retarding learning. Thus, changes in prediction errors (i.e., a decrease in the prediction that a stimulus will be followed by an event) and the ensuing decreased attention to the pre-exposed stimulus may be the mechanisms through which pre-exposure results in the LI effect.

The Hall and Rodriguez model [10] also suggests that individual differences and manipulations that increase attention during pre-exposure should result in better learning of the stimulus → no event association and, in turn, larger LI effects. Indeed, this prediction has been borne out in animal studies (e.g., [11]). In humans, pain sensitivity is one characteristic that might function to increase attention to possible predictors of pain and therefore, greater LI with pre-exposure. However, this hypothesis has not yet been tested experimentally in humans.

Using an experimental design, the current study will determine if pre-exposure retards conditioning to a noxious oral stimulus and impedes recall and retention of this learning via decreased prediction errors and attention. We will also examine whether individual differences in pain sensitivity are associated with LI of conditioned fear acquisition, recall, and retention via greater engagement of these hypothesized mechanisms.

### Hypotheses

Hypothesis 1: (a) Preexposure to a stimulus will lead to diminished prediction errors in the outcome of the pre-exposed stimulus and diminished attention to the target stimulus and (b) to decreases in fear acquisition, recall, and retention.

Hypothesis 2: Changes in prediction errors and attention will mediate the effects of preexposure on fear acquisition, recall, and retention.

Hypothesis 3: Higher pain sensitivity will be correlated with greater engagement of the targeted mechanisms and therefore, greater LI of fear acquisition, recall, and retention.

## Methods and analyses

### Participants and setting

Participant enrollment will occur at two university sites—one in northwest Ohio and one in south Texas. Based on a power analysis, we aim to recruit 100 participants from the general community, ranging in age from 6 to 35 years old. Full eligibility requirements are presented in Table 1. Participants will complete two in-person study visits and receive a $50 gift card at each visit completed.

**Table 1:** Participant eligibility criteria

Inclusion criteria	Exclusion criteria
Be between the ages of 6 and 35 years old	Currently have glasses or use contact lenses with a vision correction of ≥ ± 6
At least 2 of their maxillary anterior 6 teeth present	Are colorblind
All of their maxillary anterior 6 teeth are stable	Current injury (including fractures, open cuts, or sores) on their dominant hand
All of their maxillary anterior 6 teeth are free of cavities	Fixed dental or orthodontic appliance that would interfer with creating a mouthpiece
All of their maxillary anterior 6 teeth are free of hypersensitivity to pain or pressure	Diagnosis by a dental/oral health professional of Temporomandibular Disorder (TMD) along with a history of exacerbation of symptoms resulting from routine dental procedures
Able to read, write, and converse in English (English or Spanish at Texas site)	Currently on any anti-anxiety or anti-depressant medications
Willing and able to provide a signed and dated informed consent/assent form to participate	Currently have an inner ear infection
Willing and available to comply with all study procedures and available for the duration of the study	Living in the same household or an immediate family member of an enrolled participant
	Currently have a cardiovascular disorder or a pacemaker
	Currently have a seizure disorder or epilepsy
	History of frostbite or sensitivity to extreme cold
	Bleeding disorder or take blood thinners
	Vasospastic disorder such as Raynaud's syndrome or Raynaud's disease
	Gastrointestinal or vestibular disorders that may elevate susceptibility to nausea and dizziness such as Meniere's disease, hyperemesis gravidarum or severe migraines
	Negative outcome with VR simulation
	Medical or psychological condition that requires them to avoid mild stress
	Any medical condition that elevates risk of falls, dizziness, nausea, or causes a vasovagal reaction
	Any other general or oral health concerns

Special attempts to include Hispanic and African American participants will be made given the evidence that these populations are at greater risk for dental fear. The Ohio recruitment site will include participants from Toledo, Ohio and Detroit, Michigan. Together, these cities and the surrounding communities have a population of approximately 5 million, with approximately 22% of the population consisting of African American individuals [12]. The Texas site includes a population of 1.4 million, with 89% of the population consisting of Hispanic individuals [13].

### Study design and overview of procedures

Study variables include one measured subject variable (pain sensitivity) and two within-subjects manipulated variables [stimulus type: pre-exposed to-be conditioned stimulus (CS^+^_P_), non- pre-exposed to-be conditioned stimulus (CS_NP_^+^), and a never-conditioned stimulus (CS^−^)] and time [trial number].

After providing verbal consent, potential participants^1^ will complete a phone screen to assess eligibility. Interested participants meeting criteria will then participate in two study visits, approximately one week apart (min = 7 days,). Each visit will last approximately 90 min.

Visit 1 will begin with the completion of the consent and assent (if applicable) process, and confirmation of eligibility. Participants will then complete the measures and behavioural tasks described below as well as the experimental task. During the experimental task, the pre-exposure intervention will be administered, and fear conditioning procedures will be conducted. Immediately following, conditioned fear recall will be assessed; a minimum of 7 days later, fear retention will be assessed at Visit 2.

### Experimental task

#### Overview

**Fig. 2:**
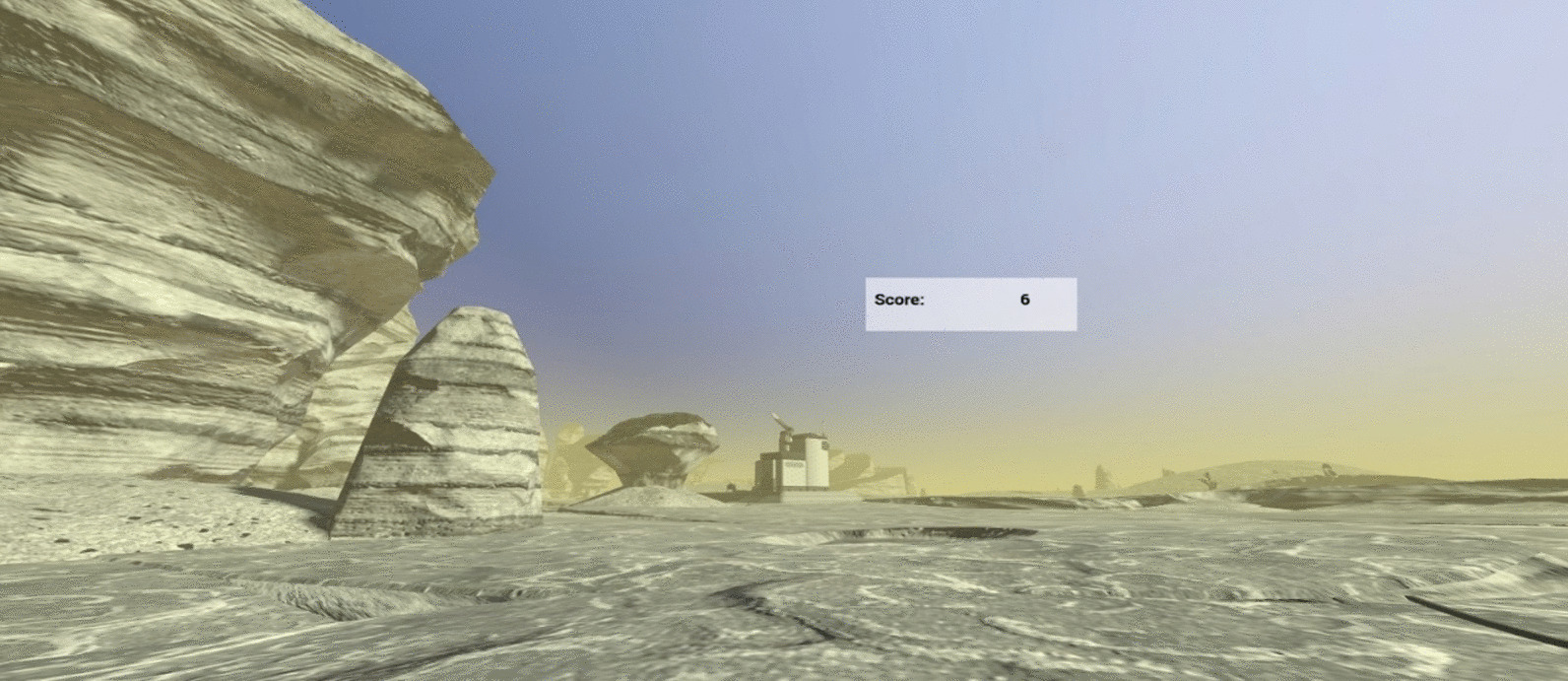
Example scene from VR task

The experimental task will be presented in virtual reality, via the HTC Vive Pro headset [14] with programming done in Vizard [15]. Use of a novel immersive environment in virtual reality allows for the control of pre-exposure (i.e., stimulus novelty) and for the assessment of approach/avoidance behavior. The task will be presented as a game in which the participant is marooned on an unfamiliar planet (see Fig. 2). The goal of the game is to collect enough fuel to return home. Participants will be told that this planet works differently from Earth, the experiences they have on the planet and with its inhabitants may not follow the same rules, and that some of the interactions on the planet may be somewhat uncomfortable. These instructions are meant to activate schemas related to pain sensitivity, paralleling experiences at a dental visit, and they also provide a rationale for the mouthpiece that the participant wears during the experimental task.

#### Stimuli

##### Aliens

Three novel aliens will serve as the CS_P_^+^, CS_NP_^+^, and CS^−^. The aliens were created for the project to ensure novelty and selected based on piloting with children, adolescents and adults which showed them to be perceived similarly and as relatively neutral; nevertheless, counterbalancing procedures will be used to rule out any stimulus-specific effects.

##### Dental startle

**Fig. 3:**
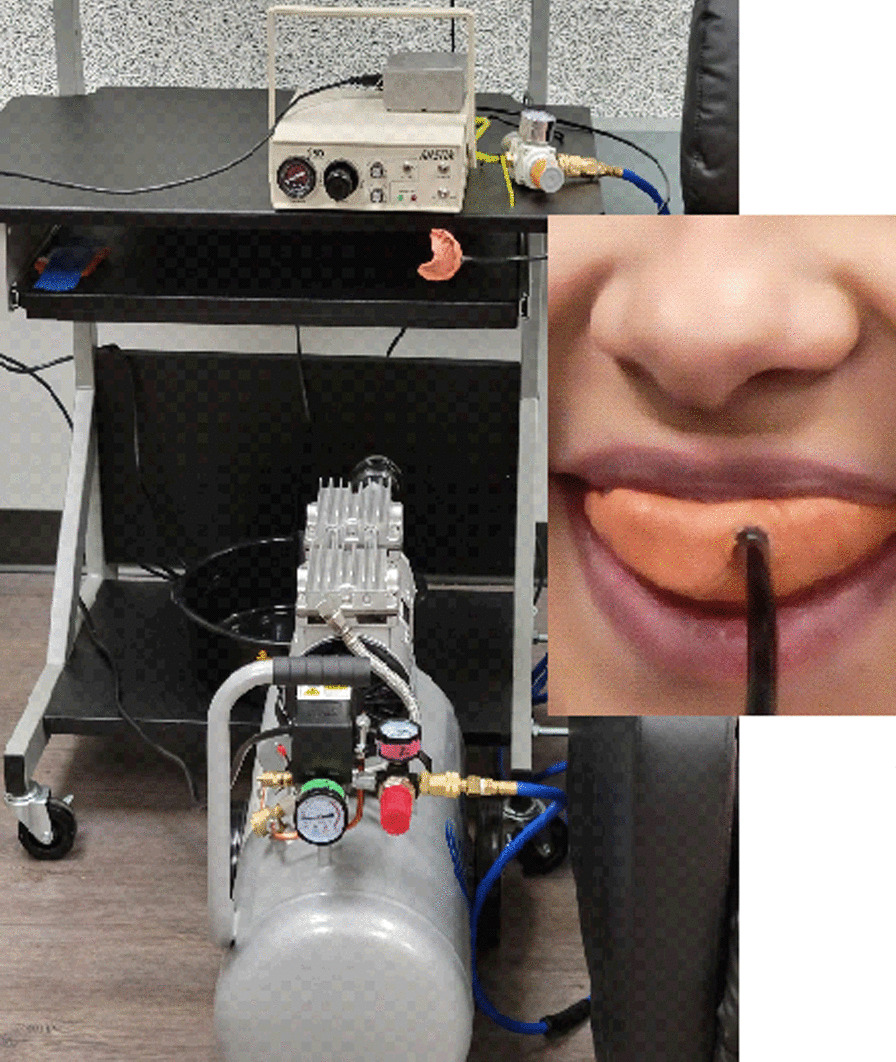
Dental startle equipment

The UCS will be a 100 ms 60 psi air puff delivered via a fitted mouthpiece, constructed with 3 M™ STD Vinyl Polysiloxane Express Putty, connected to a California Air Tools 8010 Steel Tank Air Compressor via an AIRSTIM device (San Diego Instruments, San Diego, California, USA) (see Fig. 3). Participants will be told the mouthpiece will help them to experience common sensations on the planet.

### Procedure

Although the participant will experience the “game” seamlessly, the task is broken up into four phases: pre-exposure, conditioning, and recall phases, administered during Visit 1, and a retention phase, administered during Visit 2. Each phase can be further broken down into a series of trials, which we describe next.

The *pre-exposure phase* consists of 12 trials. Each trial begins with a minimum of six seconds in which the participant is free to roam the planet using the HTC Vive Pro controllers to collect fuel hidden in the environment. At the completion of this phase of the trial an algorithm is set to deliver the CS^+^_P_, accompanied by a fuel canister, at the first opportunity.^2^ The CS^+^_P_ and the fuel canister will appear at a fixed distance from the participant. The CS^+^_P_ remains for the last six seconds of the trial. During pre-exposure, the CS^+^_P_ is never accompanied by the UCS.

The *conditioning phase* is made up of 36 trials (12 CS_P_^+^ trials, 12 CS^+^_NP_, and 12 CS^−^ trials) presented in a pseudo-random order. These trials follow the same structure as those in the pre-exposure phase with the exception that 75% of CS^+^_P_ trials, and 75% of CS^+^_NP_ are accompanied by a dental startle (described below). More specifically, the dental startle will be delivered during the last 100 ms of the trial, with the startle and alien co-terminating. The CS^−^ is never presented with the dental startle.

In the fear *recall phase* 12 trials (4 CS^+^_P_ trials, 4 CS^+^_NP_, and 4 CS^−^ trials) are presented. Again, these trials follow the structure described in the pre-exposure and conditioning phases; however, none of the aliens are presented with the UCS.

Fear retention will be assessed in the *retention phase*—a repeat of the recall phase administered a minimum of 7 days later.

### Measures

#### Candidate mediators

*Prediction errors* will be measured via participant ratings of the probability of a negative event occurring; these ratings will be selected on screen via usage of the controller and are made after the onset of the CS^+^_P_, CS^+^_NP_, or CS^−^ but prior to the onset of the UCS.

*Attention* will be measuring using eye-tracking data (i.e., dwell time).

#### Indicators of fear learning

Data on subjective, behavioral, and physical indicators of fear learning will be assessed. Participants provide ratings of their state of relaxation/anxiety when in the presence of the CS^+^_P_, CS^+^_NP_, and CS^−^ periodically throughout the experimental task. Additionally, approach behavior will be quantified in two ways; the shortest distance between the participant and the CS^+^_P_, CS^+^_NP_, and CS^−^ will be recorded on each trial as will the data on whether or not the participant collected the “fuel cell” in proximity the CS^+^_P_, CS^+^_NP_, and CS^−^. Finally, skin conductance response to the CS^+^_P_, CS^+^_NP_, and CS^−^ presentations will be recorded using two Ag–AgCl electrodermal conductance electrodes with Isotonic gel placed on the middle phalanges of the non-dominant hand ring and pinky fingers. Data from the electrodes will be recorded using Biopac MP160 with a wireless BioNomadix module transmitter (Biopac Systems, California, USA.), with a sample rate of 2000 Hz, using Biopac’s Acknowledge 5.0 software (Biopac Systems, California, USA.). Skin conductance data will be analyzed based on prior work [16].

#### Moderator

*Pain sensitivity* will be measured using a behavioral task – the cold pressor test as well a self-report measure- the Fear of Pain Questionnaire III [17] in participants 18 years of age or older and the Fear of Pain Questionnaire, Child report in participants up to age 17 years [18].

### Analyses

SPSS software will be used to address the first and third hypotheses described below; Mplus software will be used to address the second hypothesis.

Hypothesis 1a: Preexposure to a stimulus will lead to diminished prediction errors in the outcome of the pre-exposed stimulus and diminished attention to the target stimulus.

The changes expected in the candidate mediators should first be observed during the pre-exposure phase. To test hypothesis 1a effects during this phase, two separate one-way repeated measures ANOVAs (with trials during the pre-exposure phase as the factor) will be run, one with attention during pre-exposure serving as the dependent variable and the second with prediction errors during pre-exposure serving as the dependent variable. To describe the change across trials, we will examine a plot of the mean responses across trials and test for the mean change from the first to the last trial in which the mediators are assessed. The Greenhouse–Geisser adjustment will be used to address possible violations of the sphericity assumption.

To examine the effects of preexposure on the candidate mediators during the conditioning phase, attention and prediction errors captured during conditioning trials will serve as dependent variables in two separate two-way (stimulus type X trial) repeated measures ANOVAs. If a significant stimulus x trial interaction is found in these analyses, stimulus type differences will be examined at each trial (using Bonferroni-adjusted alpha), and we will examine a plot of the mean response by stimulus type and trial.

In the recall and retention phases, scores on each of the candidate mediators will be averaged across trials and a one-way within-subjects ANOVA, with stimulus type as the factor, will be conducted separately for each outcome. Significant effects will be followed-up with paired samples *t*-tests to assess for specific differences between the stimulus types.

Hypothesis 1b: Pre-exposure to a stimulus will lead to decreases in fear acquisition, recall, and retention.

Inasmuch as learning effects cannot be seen until the conditioning phase, this hypothesis is tested only during the conditioning, recall, and retention phases. To test hypothesis 1b, the conditioning phase and recall and retention phase analyses described above will be conducted with indicators of fear learning (fear ratings, approach behaviour, and skin conductance response) serving as the dependent variables. In cases in which there are multiple measurements (i.e., fear ratings, skin conductance) scores will be averaged within each phase.

Hypothesis 2: Changes in prediction errors and attention will mediate the effects of pre-exposure on fear acquisition, recall, and retention.

To test the second hypothesis, three sets of multilevel mediation models [19, 20] will be estimated to test for mediation of the fear acquisition, recall, and retention effects. Each mediation analysis will include stimulus type (dummy-coded) and the two candidate mediators—prediction errors and attention.

Parameters will be obtained by maximum likelihood estimation with robust standard errors, a procedure that is robust to violations of normality and heteroscedasticity and obtains optimal parameter estimates when response data are incomplete [21, 22], with indirect effects tested by a z test. However, given the z test may be underpowered, we will double-check results by also using Bayesian estimation, which provides for improved inference via 95% credible intervals for indirect effects [23, 24].

Hypothesis 3. Higher pain sensitivity will be associated with greater engagement of the targeted mechanisms and greater LI of conditioned fear acquisition, recall, and retention.

To test the third hypothesis, individual differences in pain sensitivity will be included in the above analyses as a moderating variable.

## Discussion

This protocol describes the design of a study to test hypotheses regarding the mechanisms through which pre-exposure interventions result in LI of conditioning to a noxious oral stimulus. Additionally, the ability of a relevant individual difference variable, pain sensitivity, to predict the magnitude of LI via engagement of the hypothesized mechanisms will be tested. Results from this study could be used to design prevention programs for dental phobia and to predict for whom these programs will be most effective.

A practical issue that could affect study quality include the possibility that assessment of the variables of interest through participant ratings could either facilitate or interfere with the learning taking place in the conditioning task and/or the physiological assessment of fear learning. There is some evidence, however, that these effects can be minimized or eliminated through intermittent (i.e., not on every trial) ratings and careful timing of the rating and physiological measurements [25]; therefore, that is the approach utilized here. A second possible issue is the reliable execution of study procedures across the two study sites. Given that the study intervention is automated using an identical VR program at both sites, the concerns here are greatly minimized. Detailed standard operating procedures and training requirements will also be used to maximize fidelity.

## Data Availability

Not applicable.

## Abbreviations

ANOVA: Analysis of variance
CS^−^: Never-conditioned stimulus
CS^+^_NP_: Non-pre-exposed to-be conditioned stimulus
CS^+^_P_: Pre-exposed to-be conditioned stimulus
LI: Latent inhibition
ms: Millisecond
psi: Pounds per square inch
UCS: Unconditioned stimulus

## Footnotes

1 In the case of children or adolescents, screening and screening consent will be done with a parent or legal guardian.

2 The algorithm is set to display an alien six seconds into the trial; however, the presentation of the alien is delayed if the participant is facing a structure that would not allow the alien to appear in the correct position, creating a variable inter-trial interval.
